# Between‐hospital variation in rates of complications and decline of patient performance after glioblastoma surgery in the dutch Quality Registry Neuro Surgery

**DOI:** 10.1007/s11060-021-03697-8

**Published:** 2021-01-28

**Authors:** Ivar Kommers, Linda Ackermans, Hilko Ardon, Wimar A. van den Brink, Wim Bouwknegt, Rutger K. Balvers, Niels van der Gaag, Lisette Bosscher, Alfred Kloet, Jan Koopmans, Mark ter Laan, Rishi Nandoe Tewarie, Pierre A. Robe, Olivier van der Veer, Michiel Wagemakers, Aeilko H. Zwinderman, Philip C. De Witt Hamer

**Affiliations:** 1 Department of Neurosurgery, Location VUmc, Cancer Center Amsterdam, Amsterdam University Medical Centers, De Boelelaan 1117, 1081 HV Amsterdam, Netherlands; 2 Department of Neurosurgery, Maastricht University Medical Center, Maastricht, Netherlands; 3 Department of Neurosurgery, St Elisabeth Hospital, Tilburg, Netherlands; 4 Department of Neurosurgery,, Isala, Zwolle, Netherlands; 5 Department of Neurosurgery, Medical Center Slotervaart, Amsterdam, Netherlands; 6 Department of Neurosurgery, Erasmus University Medical Centre, Rotterdam, Netherlands; 7 Department of Neurosurgery, Northwest Clinics, Alkmaar, Netherlands; 8 Department of Neurosurgery, Medical Center Haaglanden, The Hague, Netherlands; 9 Department of Neurosurgery, Martini Hospital, Groningen, Netherlands; 10 Department of Neurosurgery, Radboud University Medical Center, Nijmegen, Netherlands; 11 Department of Neurosurgery, Leiden University Medical Center, Leiden, Netherlands; 12 Department of Neurology & Neurosurgery, University Medical Center Utrecht, Utrecht, Netherlands; 13 Department of Neurosurgery, Medical Spectrum Twente, Enschede, Netherlands; 14 Department of Neurosurgery, University Medical Center Groningen, Groningen, Netherlands; 15 Department of Clinical Epidemiology and Biostatistics, Amsterdam University Medical Centers, Amsterdam, Netherlands

**Keywords:** Glioblastoma, Neurosurgical procedures, Postoperative complications, Karnofsky performance status, Quality of health care, Patient outcome assessment

## Abstract

**Introduction:**

For decisions on glioblastoma surgery, the risk of complications and decline in performance is decisive. In this study, we determine the rate of complications and performance decline after resections and biopsies in a national quality registry, their risk factors and the risk-standardized variation between institutions.

**Methods:**

Data from all 3288 adults with first-time glioblastoma surgery at 13 hospitals were obtained from a prospective population-based Quality Registry Neuro Surgery in the Netherlands between 2013 and 2017. Patients were stratified by biopsies and resections. Complications were categorized as Clavien-Dindo grades II and higher. Performance decline was considered a deterioration of more than 10 Karnofsky points at 6 weeks. Risk factors were evaluated in multivariable logistic regression analysis. Patient-specific expected and observed complications and performance declines were summarized for institutions and analyzed in funnel plots.

**Results:**

For 2271 resections, the overall complication rate was 20 % and 16 % declined in performance. For 1017 biopsies, the overall complication rate was 11 % and 30 % declined in performance. Patient-related characteristics were significant risk factors for complications and performance decline, i.e. higher age, lower baseline Karnofsky, higher ASA classification, and the surgical procedure. Hospital characteristics, i.e. case volume, university affiliation and biopsy percentage, were not. In three institutes the observed complication rate was significantly less than expected. In one institute significantly more performance declines were observed than expected, and in one institute significantly less.

**Conclusions:**

Patient characteristics, but not case volume, were risk factors for complications and performance decline after glioblastoma surgery. After risk-standardization, hospitals varied in complications and performance declines.

**Supplementary Information:**

The online version contains supplementary material available at 10.1007/s11060-021-03697-8.

## Introduction

For patients with glioblastoma, neurosurgeons aim to maximize tumor removal, while preserving functional integrity, to prolong patient survival with acceptable quality of life [1]. Based on various factors, including age, symptoms, general condition, comorbidity, tumor location and extent, perceived balance between procedural risks and anticipated benefit and patient preference, the decision is sometimes made to biopsy rather than to resect, and to limit the extent of resection. An important argument to make neurosurgical decisions and to counsel patients is the risk of complications and a decline in performance.

Standards are lacking for the indication to biopsy and for the extent of tumor removal. Consequently a large range of options is available for neurosurgical teams which can be considered an opportunity for highly patient-tailored decisions, but at the same time could result in considerable practice variation and therefore outcome variation. We have previously reported on variation in 30-day mortality and 2-year survival outcome among institutes from the same registry [2].

The literature contains surgical reports with varying rates of adverse events after glioblastoma surgery. Instead of selectively citing the rate that suits the present decision best, it is probably better to use real-world data as source for arguments in these neurosurgical decisions, preferably using one’s own outcome data. To this end the Dutch Society for Neurosurgery has initiated a quality registry, which contains outcome data from all patients who had glioblastoma surgery in all institutes.

In this study, we determine the rate and severity of complications and Karnofsky performance decline after resections and biopsies in a nation-wide quality registry, their risk factors and the risk-standardized variation between institutions.

## Materials and methods

### Dutch quality registry neuro surgery

The Dutch Society for Neurosurgery (http://www.nvvn.org) established the Quality Registry for Neuro Surgery in 2011 (http://www.qrns.nl). This registry aims to provide feedback to all institutions with neurosurgical units on patient outcomes and treatment variation for self-assessment and quality-monitoring. Neurosurgeons, nurse specialists in neuro-oncology and trained physician assistants prospectively enter patient data in the registry. Participation in the registry is mandatory for all intitutions providing glioblastoma surgery. Each institution is represented in the collaborative for glioblastoma surgery with several meetings per year for the methods design and interpretation of results. Outcomes are reported to the Dutch Society for Neurosurgery annually.

### Patients

We studied all 3288 patients who had first-time surgery for glioblastoma at all 13 hospitals in the Netherlands. Patients had their surgery between 1/1/2013, when the complication severity was included, and 12/31/2017. We collected data for patients 18 years or older at surgery and a histopathological diagnosis of glioblastoma according to the WHO 2007 criteria until 2015 and the WHO 2016 criteria thereafter [3].

### Data collection

Demographic and clinical information consisted of age at diagnosis, gender, Karnofsky performance status before surgery, type of surgery (biopsy or resection), and dates of treatment, last follow-up and death. A surgical procedure was considered a biopsy, when tissue was taken for diagnosis only, either by needle biopsy or open biopsy.

Treatment decisions for patients were made in multidisciplinary tumor board meetings in all hospitals. Image-guided navigation was available in all hospitals, fluorescence-guidance in ten hospitals, intraoperative stimulation mapping in nine hospitals, ultrasound in three hospitals, and intraoperative MRI in none of the hospitals.

Because this data was collected for evaluation of quality of care in accordance with the Dutch Quality Act for Healthcare (http://wetten.overheid.nl/BWBR0007850/2015-01-01) and the New Healthcare Quality, Complaints and Disputes Act (https://wetten.overheid.nl/BWBR0037173/2019-05-01), individual written informed consent was not needed. The study was not subject to the Medical Research Involving Human Subjects Act (WMO, https://wetten.overheid.nl/BWBR0009408/2018-08-01), therefore ethical approval was waived, and de-identified data had been collected of patients not alive by a trusted third party (http://www.sivz.nl/en).

### Outcome measures and risk predictors

The main outcome measures to evaluate variation were specified in the consensus item set: the risk-standardized complications and performance alterations at 6 weeks postoperative. The severity of complications was graded by the revised Clavien-Dindo classification. This classification ranks complications based on the therapy used to treat the complication and has been reported to be an objective, simple, reliable, and reproducible way of reporting adverse events after surgery [4], and consists of five grades, i.e. I: any deviation from normal, not requiring treatment, II: requiring medication, III: requiring an intervention, IV: requiring intensive care management, and V: death. This grading was added to the registry in 2013. For institutional comparison, we analyzed complication severity as complications of grade II and higher. Performance alterations were calculated by subtracting the baseline Karnofsky performance score prior to surgery from the Karnofsky performance score at 6 weeks after surgery. For institutional comparison, negative performance change of more than ten points was considered a performance decline [5].

To account for risk differences between institutions, we explored these patient-related characteristics as predictors for outcomes: age at surgery, gender, American Society of Anesthesiologists physical status (ASA) classification, baseline Karnofsky performance, and year of treatment; and the institution-related characteristics: case volume, university hospital, and biopsy percentage.

### Statistical analysis

For each patient a risk prediction was calculated based on the patient characteristics that were identified in multivariable logistic regression models. The model with the lowest Akaiki’s information criterium was selected as a trade-off between goodness of fit and model simplicity. Patient-specific risk prediction allowed for risk-standardized comparison between expected and observed complication grades and performance decline for institutes. To compare outcomes between institutes, the expected number of events based on an institute’s patient population was plotted against the ratio of number of observed and expected events in funnel plots. Institutes with achievements outside the 95 % confidence intervals were considered significantly deviant from the expectation. Institutes, providing less than 85 % of outcome measures, were considered to contribute insufficient data and their results were therefore uninformative.

## Results

Of the 3288 patients, 2271 (69 %) had a resection, the others a biopsy only. The complication severity was missing for 417 (13 %). A baseline or follow-up performance score to calculate the performance change was missing for 440 (13 %). This was mainly due to 3 of 13 institutes, f, h, and i, with more than 15 % of outcome measures missing. Patient characteristics per institute and institutional characteristics are listed in Table 1.

**Table 1:** Characteristics of patients and hospitals with complication grading and performance decline per institute and overall

Institute	a	b	c	d	e	f	g	h	i	j	k	l	m	Overall
Number of patients	80	112	119	120	181	197	236	237	335	387	413	422	449	3288
Number of complete cases for complication grading	79	112	104	115	157	7	229	144	256	384	413	422	449	2871
Percentage of complete cases for complication grading	99 %	100 %	87 %	96 %	87 %	4 %	97 %	61 %	76 %	99 %	100 %	100 %	100 %	87 %
Number of complete cases for performance changes	80	112	119	112	173	52	235	126	234	383	357	421	444	2848
Percentage of complete cases for performance changes	100 %	100 %	100 %	93 %	96 %	26 %	100 %	53 %	70 %	99 %	86 %	100 %	99 %	87 %
Patient-related characteristics														
Number of females	28	52	49	39	65	79	89	96	124	127	149	163	162	1222
Percentage of females	35 %	46 %	41 %	33 %	36 %	40 %	38 %	41 %	37 %	33 %	36 %	39 %	36 %	37 %
Mean age	61.1	61.3	62.8	62.2	61.4	59.5	60.6	60.7	60.1	57.7	62.0	62.9	66.1	61.6
s.d. of age	10.9	11.6	11.0	12.8	11.1	15.2	12.8	14.0	11.8	14.2	11.3	13.3	90.2	35.8
ASA classification														
ASA I	46	24	28	12	33	40	42	46	123	141	150	51	77	813
ASA II	28	61	64	83	105	88	154	133	108	191	215	305	199	1734
ASA III	5	23	5	18	25	15	38	25	75	51	43	57	52	432
ASA IV	1	1	0	1	1	0	2	1	2	0	3	6	6	24
ASA V	0	0	1	0	1	1	0	0	0	1	0	2	0	6
ASA missing	0	3	21	6	16	53	0	32	27	3	2	1	115	279
Preoperative Karnofsky performance score														
KPS 100	8	2	19	16	11	34	14	8	33	83	53	24	23	328
KPS 90	36	32	48	38	61	68	60	79	96	126	193	154	124	1115
KPS 80	12	24	29	13	45	29	65	62	90	72	87	136	107	771
KPS 70	4	24	11	18	29	29	28	25	59	53	20	52	97	449
KPS 60	6	18	5	18	19	6	32	18	32	32	9	35	58	288
KPS 50	8	11	4	8	14	2	29	13	11	17	7	16	23	163
KPS 40	3	1	0	3	1	1	2	4	4	1	1	2	5	28
KPS 30	1	0	0	0	0	1	3	0	1	2	1	1	3	13
KPS 20	2	0	2	0	1	1	3	7	2	1	0	1	3	23
KPS 10	0	0	1	0	0	0	0	0	2	0	0	1	1	5
KPS missing	0	0	0	6	0	26	0	21	5	0	42	0	5	105
Year of treatment														
2013	22	26	27	27	23	37	48	37	54	59	81	76	87	604
2014	16	25	15	20	47	47	46	49	74	79	88	77	75	658
2015	12	22	18	25	35	44	45	56	88	67	80	81	100	673
2016	22	22	28	27	33	31	50	36	89	76	99	87	83	683
2017	8	17	31	21	43	38	47	59	30	106	65	101	104	670
**Hospital-related characteristics**														
Academic setting	No	No	No	No	Yes	Yes	Yes	No	Yes	Yes	No	Yes	Yes	7
Number of patients with biopsy	20	33	49	36	61	130	57	45	72	99	164	106	145	1017
Percentage of patients with biopsy	25 %	29 %	41 %	30 %	34 %	66 %	24 %	19 %	21 %	26 %	40 %	25 %	32 %	31 %
Number of patients with complication grade II–V	2	15	3	13	13	1	39	19	28	22	39	45	58	297
Percentage of patients with complication grade II–V	3 %	13 %	3 %	11 %	7 %	1 %	17 %	8 %	8 %	6 %	9 %	11 %	13 %	9 %
Number of patients with performance decline	15	8	38	24	51	4	47	19	63	72	74	129	120	664
Percentage of patients with performance decline	19 %	7 %	32 %	20 %	28 %	2 %	20 %	8 %	19 %	19 %	18 %	31 %	27 %	20 %

**Fig. 1:**
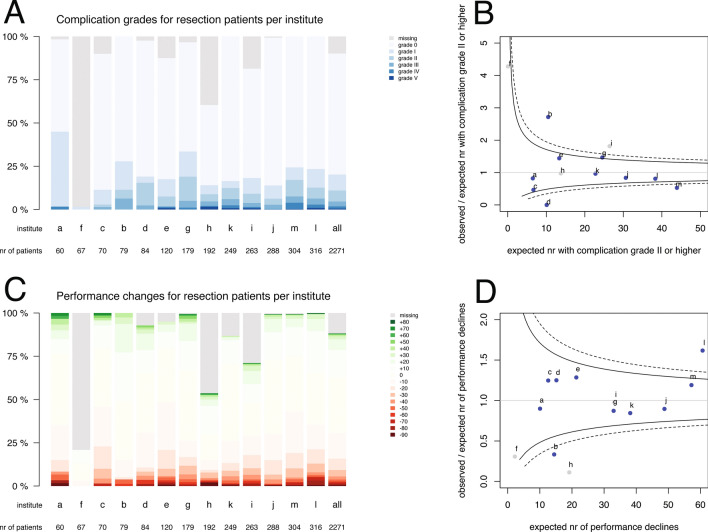
Distributions of **a** Clavien-Dindo complication severity and **c** Karnofsky performance changes for resection patients per institute, sorted by volume of **resection** cases over 5 years and funnel plots for observed and expected **b** complication grade II or higher and **d** performance decline. The color codes are provided in the legends. Each dot represents an institute indicated by a letter corresponding to Table 1. Blue dots indicate institutes with less than 15 % outcome measures missing, grey dots institutes with more than 15 % missing. The solid funnels are 95 % control limits, the dotted funnels 99 % control limits

**Fig. 2:**
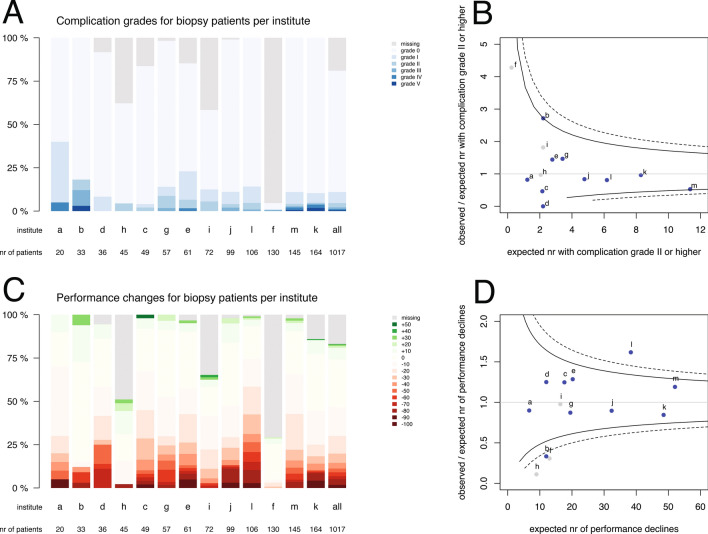
Distributions of **a** Clavien-Dindo complication severity and **c** Karnofsky performance changes for biopsy patients per institute, sorted by volume of **biopsy** cases over five years and funnel plots for observed and expected **b** complication grade II and higher and **d** performance decline. The color codes are provided in the legends. Each dot represents an institute indicated by a letter corresponding to Table 1. Blue dots indicate institutes with less than 15 % outcome measures missing, grey dots institutes with more than 15 % missing. The solid funnels are 95 % control limits, the dotted funnels 99 % control limits

The observed complication severity and performance changes at 6 weeks postoperative are plotted in Fig. 1 for resections and in Fig. 2 for biopsies. For resections over all institutes, complications of any severity were observed in 459 (20 %) patients, grade II or higher in 250 (11 %), grade III or higher in 105 (5 %) and grade IV or higher in 41 (2 %) (Fig. 1a). Complications of grade II or higher for resections ranged between 0 % (institute f) and 19 % (institute g). And a performance decline was observed in 359 (16 %) resection patients, a stable performance in 1427 (63 %), and a performance improvement in 217 (10 %) (Fig. 1c). Performance decline for resections varied from 0 % (institute f) to 23 % (institute c). For biopsies over all institutes, complications of any grade were observed in 112 (11 %) patients, grade II or higher in 47 (5 %), grade III or higher in 22 (2 %) and grade IV or higher in 12 (1 %) (Fig. 2a). Complications of grade II or higher for biopsies varied from 0 % (institute d) to 18 % (institute b). And a performance decline was observed in 305 (30 %) biopsy patients, a stable performance in 518 (51 %), and a performance improvement in 22 (2 %) (Fig. 2c). Performance decline for biopsies ranged between 2 % (institute h) and 58 % (institute l).

To determine the risk factors for complication severity and performance decline, we first plotted the patient and the institute characteristics to these outcomes (Supplementary Fig. 1). Next, the association between characteristics and a complication grade II or higher, or a performance decline was evaluated in multivariable logistic regression models. A higher risk of a complication grade II or higher was associated with lower baseline Karnofsky (odds ratio, 95 % confidence interval: 0.97, 0.97–0.98), a higher ASA classification (ASA-II to ASA-I: 1.6, 1.1–2.2; ASA-III to ASA-I: 1.8, 1.2–2.8; ASA-IV to ASA-I: 3.0, 1.0-7.9; ASA-V to ASA-I: 1.2, 0.1–11), and a resection (compared to a biopsy: 2.7, 1.9–3.8). This model has an AIC of 1774 and the interaction terms were not significantly associated. Year of treatment, patient age and gender were not associated with a complication grade II or higher. A higher risk of performance decline of more than 10 points was associated with a higher age (1.02, 1.01–1.03), higher ASA classification (ASA-II to ASA-I: 1.2, 0.97–1.6; ASA-III to ASA-I: 1.6, 1.1–2.2; ASA-IV to ASA-I: 2.6, 1.0-6.3; ASA-V to ASA-I: 5.8, 0.93-45) and a biopsy (compared to a resection: 2.3, 1.9–2.7). This model has an AIC of 2922 and a significant interaction term between age and ASA classification. Year of treatment and gender were not associated with performance decline. Of note, the institution characteristics overall case volume, university hospital and biopsy percentage were not associated with complication severity nor with performance decline (Supplementary Figs. 1 and 2).

**Fig. 3:**
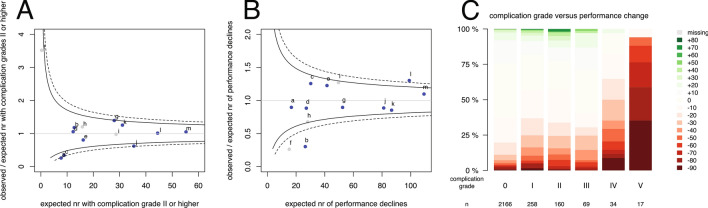
Funnel plot for all patients per institute of risk-standardized expected number of events and the ratio of observed and expected number of events for **a** a complication grade II and higher, and **b** for a performance decline of more than ten points. Each dot represents an institute indicated by a letter corresponding to Table 1. Blue dots indicate institutes with less than 15 % outcome measures missing, grey dots institutes with more than 15 % missing. The solid funnels are 95 % control limits, the dotted funnels 99 % control limits. Correlation between complication grades and performance changes (**c**)

The between-institution variation in complication severity and performance decline is displayed as funnel plot for all patients in Fig. 3a, b. Ratios higher than 1.0 indicate more adverse events than expected based on risk standardization. In three institutes, a, c, and j, the number of observed patients with complications was significantly less than expected (Fig. 3a). In institute l significantly more performance declines were observed than expected, and in b significantly less (Fig. 3b). Other institutions had ratios within the control limits, i.e. observed events were according to expectations. As the type of intervention was a strong risk factor for outcomes, funnel plots were generated separately for the subgroup with a resection (Fig. 1b, d) and for the subgroup with a biopsy (Fig. 2b, d).

The correlation between Clavien-Dindo classification and the change in Karnofsky performance was low (Kendall’s tau correlation: − 0.14, Fig. 3c). No complication was observed in 198 (55 %) of 359 resection patients with a performance decline and in 228 (75 %) of 305 biopsy patients with a performance decline. Conversely, a performance improvement was observed in 109 (45 %) of 250 resection patients with a complication grade II or higher and in 7 (13 %) of 47 biopsy patients with a complication grade II or higher.

## Discussion

The main findings of this study are: (1) any complication is observed in 20 % after resection and in 11 % after biopsy; a performance decline was observed in 16 % after resection and in 30 % after biopsy, (2) risk factors for a complication were lower baseline Karnofsky, higher ASA classification, and a resection; risk factors for a performance decline were higher age, higher ASA classification and a biopsy; institutional case volume, biopsy percentage and university hospital were not associated with complications nor performance decline, (3) patient outcomes among institutes vary more in complications than in performance decline.

Variation between institutions in complication outcomes and performance changes has not been published for glioblastoma surgery. Compared to the extensive literature on benefits of glioblastoma surgery, the literature on adverse outcome is limited. In these reports, the definitions of surgical complications, the classifications of patient condition and the timing of assessment are far from uniform. Complications after glioblastoma resection varied between 15 % [6], 19 % [7], 23 % [8], 24 % [9], and 68 % [10]. Complications of biopsies varied between 3 % [11], 6 % [12], 7 % [13], 8 % [14], 9 % [15] ,12 % [16], and 13 % [17]. We now demonstrate the outcome variation in adverse events after glioblastoma surgery among teams using identical definitions and risk-standardization.

Some reports have used the Karnofsky performance score. The median Karnofsky performance before and after surgery were reported as similar [18–21]. The performance change is more informative for risk assessment in individual patients. For instance, a performance decline was observed in 5 % [22], 10 % [23], and 39 % [7].

Other measures of adverse outcome of glioblastoma surgery have been documented. Several reports narrow down patient condition to neurologic outcome [24–26]. Yet, others have used readmission rate as surrogate marker [6]. Even more scarce are reports on health-related quality of life after surgery [27, 28].

Apart from divergent complication definitions as a source of variation in the literature, the timing of assessment also varied: 21 days [9], 30 days [8, 23], 6 weeks [7], 3 months [26], and 6 months after surgery [24]. Too early assessment would inadvertently include transient neurologic dysfunctions and exclude late complications from surgery. Too late assessment would include decline from tumor progression or adverse events from other treatments. Therefore, we selected 6 weeks after surgery, typically immediately before the start of radiotherapy.

To compare complication outcomes between neurosurgical reports, a consensus definition of complications, their severity and their timing of assessment is essential. Few classifications have been proposed [29–32]. Ambiguity arises in classifying adverse events when judgment of deviation from the expected course is required. We have chosen to use the revised Clavien-Dindo classification, because it avoids this ambiguity from expectations and allows for direct comparison with other surgical procedures. In general surgery this has been proven to be an objective, simple, reliable, and reproducible way of reporting adverse postoperative events [4, 33]. This classification is based on the type of therapy required to treat the complication and was devised to eliminate subjective interpretation of serious adverse events, because it is based on events that are usually well-documented and easily verified. To put the observed complication risk of glioblastoma surgery into perspective, any complication was observed in 10 % after radical prostatectomy [34], 29 % after hepatocellular carcinoma resections [35], 29 % after noncardiac thoracic operations [36], 47 % after pancreatic adenocarcinoma resections [37], and 61 % after pancreaticoduodenectomies [38].

The Clavien-Dindo classification and the change in Karnofsky performance can both detect adverse surgical events, although these measure different aspects of patient outcome and their correlation was low. Others have observed more performance decline in patients with higher grade complications in a general neurosurgical population [30, 39]. An explanation for the discordance between the two measures in our data may be that post-operative neurological deficit is not scored in the Clavien-Dindo classification, when it does not require additional treatment. Another explanation may be that not every performance decline is due to surgical complications, but can also be due to early glioblastoma progression. We have previously identified early progression as frequent cause of death within 30 days [2]. Early progression may also explain the discordance between 30 % performance decline after biopsy and 16 % decline after resection.

The implications for clinical practice from this work are that the risk factors for complications, i.e. lower baseline Karnofsky and higher ASA classification, can be used for patient counseling. For example, consider two patients indicated for a resection: a 75-year-old patient with a KPS of 70, and an ASA classification of III, and a 25-year-old patient with a KPS of 90, and an ASA classification of I. The first patient has a risk of 18 % for a relevant complication and 24 % for performance decline, whereas the second patient 7 % and 14 %, respectively (see also https://nvvn-qrns-gbm.shinyapps.io/patient_risk_prediction/). Ideally, patient counseling should be based on institute-specific data, which is now available for these hospitals. Others have identified higher age and tumor location as risk factors for complications [10, 23]. The systematic collection of imaging characteristics has been scheduled for our registry from 2020 to be able to evaluate tumor volume and location as determinant of patient outcome in future analyses.

The discussions based on these results in the registry collaborative and in the institutions have been constructive and should contribute to outcome improvement programs for all institutes. Nevertheless this improvement has yet to be determined. A recent systematic review on the effectiveness of quality improvement collaboratives showed a statistically significant improvement of at least 50 % of the primary outcome in 73 % of studies [40]. The elements for success from collaboratives have not been identified [41, 42]. The aim for our collaborative on glioblastoma surgery is to further reduce complications and performance decline, rather than facilitate regression to a common mean outcome with no outliers. We will expand the registry with more detailed neurological outcome and complication diagnosis to be able to address potential strategies for improvement.

Strengths of this study include a comprehensive population-based nation-wide prospective registry with standardized definitions of severity and timing of complications and performance decline. As limitations, we did not specify the neurological outcomes, the complication diagnosis and potential causes and we did not measure the health-related quality of life. Imaging data including tumor volume and location was so far not systematically collected and consequently risk-standardization could be improved. Some treatment-related characteristics that may be of interest as predictors were not systematically collected in the registry, such as corticosteroid use, surgical technique and extent of resection. Data verification by audits could support the data quality.

## Conclusions

Any complication in glioblastoma surgery is observed in 11 % after biopsy and in 20 % after resection, and a performance decline was observed in 30 % after biopsy and in 16 % after resection. The risk factors for a complication were lower baseline Karnofsky, higher ASA classification, and a resection. The risk factors for a performance decline were higher age, higher ASA classification and a biopsy. Institution case volume, academic status and biopsy percentage were not associated with complications nor performance decline. Complications and performance declines vary between hospitals.

## Supplementary Informatiom

Below is the link to the electronic supplementary material.

Supplemental Figure 1 Boxplots of patient and hospital characteristics versus complication grades and performance changes. Supplementary Material 1 (TIF 7373 kb)

Supplemental Figure 2 Scatterplots of hospital characteristics versus complication grades and performance changes. Each dot represents an institution with corresponding identification. Blue dots indicate institutes with less than 15 % outcome measures missing, grey dots institutes with more than 15 % missing. The solid dots indicate university hospitals, the open dots indicate general hospitals. The solid black lines plot the estimated regression coefficients, and the dotted black lines represent the 95 % confidence intervals of the regression coefficients. The solid grey line represents the overall population percentage of complications grade 2 and higher, and percentage of performance declines over 20 points. Supplementary Material 2 (TIF 1658 kb)
